# *treNch*: Ultra-Low Power Wireless Communication Protocol for IoT and Energy Harvesting

**DOI:** 10.3390/s20216156

**Published:** 2020-10-29

**Authors:** Fernando Moreno-Cruz, Víctor Toral-López, Antonio Escobar-Molero, Víctor U. Ruíz, Almudena Rivadeneyra, Diego P. Morales

**Affiliations:** 1 Infineon Technologies AG, 85579 Neubiberg, Germany; Antonio.Escobar@infineon.com; 2 Department of Electronics and Computer Technology, University of Granada, 18004 Granada, Spain; vtoral@ugr.es (V.T.-L.); arivadeneyra@ugr.es (A.R.); diegopm@ugr.es (D.P.M.); 3 Eesy-Innovation GmbH, 82008 Unterhaching, Germany; ruiz.quero@eesy-innovation.com

**Keywords:** Wireless Sensor Networks (WSN), Internet of Things (IoT), ultra-low power, Bluetooth Low Energy (BLE), energy harvesting

## Abstract

Although the number of Internet of Things devices increases every year, efforts to decrease hardware energy demands and to improve efficiencies of the energy-harvesting stages have reached an ultra-low power level. However, no current standard of wireless communication protocol (WCP) can fully address those scenarios. Our focus in this paper is to introduce *treNch*, a novel WCP implementing the cross-layer principle to use the power input for adapting its operation in a dynamic manner that goes from pure best-effort to nearly real time. Together with the energy-management algorithm, it operates with asynchronous transmissions, synchronous and optional receptions, short frame sizes and a light architecture that gives control to the nodes. These features make *treNch* an optimal option for wireless sensor networks with ultra-low power demands and severe energy fluctuations. We demonstrate through a comparison with different modes of Bluetooth Low Energy (BLE) a decrease of the power consumption in 1 to 2 orders of magnitude for different scenarios at equal quality of service. Moreover, we propose some security optimizations, such as shorter over-the-air counters, to reduce the packet overhead without decreasing the security level. Finally, we discuss other features aside of the energy needs, such as latency, reliability or topology, brought again against BLE.

## 1. Introduction

The eruption of the Internet of Things (IoT) in our society has presented us with a scenario where every object is connected to the Internet for interaction. In this heterogeneous infrastructure, each intelligent point can communicate with the rest of the nodes, services or people through different topologies to substantially affect the user’s experience and quality of life.

Under those circumstances, a challenge arises when powering the nodes without affecting the application quality of service (QoS). Generally, a power cord is excluded from the possible options because of its expenses, inconvenience or impracticability [1]. A common option is batteries; however, not only their cost in terms of their frequent replacement or recharge, but also their environmental impact play an important role: non-rechargeable chemical batteries (e.g., lithium) are a common choice, which are deeply contaminating [2]. Within this framework energy harvesting (EH) appears as a way of taking advantage of the diverse energy present in the environment, converting it into electricity for its use.

**Figure 1:**
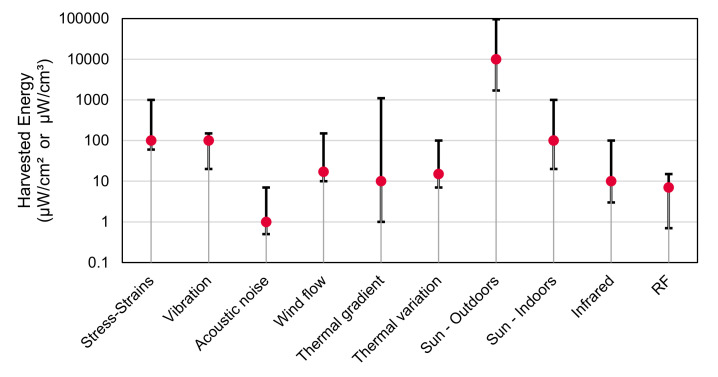
Power comparison of most common EH sources [3]. Typical range and most frequent value.

EH, however, encounters some difficulties in terms of the power capable of being extracted and its stability over time. Most of the EH sources are categorized as “ultra-low power (ULP)” (barely tens of microwatts) [3], see Figure 1. Moreover, the energy flow might unpredictably rise, drop, or directly stop, e.g., weather changes in solar, wind and thermal or lack of band use in RF. These force the applications over these technologies to be ULP as well, in addition to not presenting extremely low latency or high-dependability demands.

To address this, two fronts emerge:

1. Hardware, where every stage must be optimized. Starting from efficiency of energy gathering and conversion phases [4,5,6,7], and ending with consumption of the microcontroller (µC) and transceiver.
2. Software, where the operating system (OS) and the wireless communication protocol (WCP), often together, have the greatest influence. They are light and simple, and follow the ULP requirements without compromising the QoS [8,9,10].

An ULP IoT application generally operates in a duty-cycled fashion. This means remaining in a low energy consumption state (sleep phase) during most of the time and waking up to carry out measurements and communication (active phase). In recent years, a lot effort has been devoted to the improvement of the µCs and radio transceiver performance. Outstanding consumption figures have been achieved of less than 300 nW and 1 mJ for the sleep and active phases, respectively [5].

Meanwhile, low and ULP WCPs have emerged and extensively been deployed, although most of them are not suitable for EH use cases. Their operating principles and architectures do not always match these scarce energy profiles, often also burdened by legacy modes and the pursuit of broad interoperability. In particular, they often employ large packet-headers that lead to unnecessary communication overhead [11], normally due to the desire for more functionality or compatibility that other use cases can energetically afford, i.e., WCPs that first spotlight more energy-advantageous scenarios. Likewise, the bases of their operation, founded frequently on synchronous communications, imply the use of power consuming timers during the sleep phase or of the radio by the medium-sensing in asynchronous cases that make them more energy-demanding. Moreover, the cycle times are determined without considering the energy status, leading to more failures and therefore inefficiency [12]. The reception at the nodes, for its part, which represents a great sink of energy [13], is habitually realized indiscriminately, despite its necessity. Moreover, even the WCPs with ULP requirements are often designed to work with a constant power source, i.e., a battery, and are not efficient with abundant power drops, i.e., an EH supply [12].

### 1.1. Related Work

Table 1 summarizes the most extended WCPs for embedded systems and ultra-/low power use cases. Bluetooth Low Energy (BLE), after its last release in 2017, stands out for the ULP scenarios [14]. Its functionality without previous connection (advertising) and the more recent mesh option has achieved extraordinarily low energy requirements. The advertising mode, however, lacks the reception feature and does not count with security (besides some connection-based modes having weaknesses as well) [15]. In the mesh case, an ULP node needs a “friend” node for implementing receptions and security, which brings overhead and also vulnerabilities [16,17]. Solving this would require the establishment of a traditional master–slave connection with synchronous operation, leaving behind its benefits. Zigbee and Thread, both based on the IEEE 802.15.4 standard, exhibit excellent power consumption as well, and are a possible solution for the most energetic EH use cases. ANT and Z-Wave, besides being proprietary protocols, stand already on slightly higher energy levels, as does Wi-Fi, intended for higher data rates and not fitting in these ULP scenarios. EnOcean, on the other side, is the only one decidedly designed for EH, although with limited functionality and of proprietary use, which complicates its deployment.

Other solutions not considered are NB-IoT (5G), LoRaWAN and Sigfox, as they are designed for long-range communications (WWAN) and have higher power consumptions. RFID (radio frequency identification) and near field communication (NFC) stay out of the scope as well, since they need express interaction for every transmission, as well as being proximity communication protocols.

Concerning the power management, the state-of-the-art WCPs address it with different energy-saving techniques, which make them fit with different use cases; see Table 1. Presuming duty-cyclic functionality for all of them, the trend is asynchronous transmissions, where the node decides when to wake up and transmit. However, some protocols also implement it synchronously, such as BLE (in its connection-based mode), with time slots, as WirelessHart (based on IEEE 802.15.4 too) [29], or even hybrid modes between them, such as ANT or Zigbee.

Furthermore, the physical layer plays an important part. Low data rates and FSK- and PSK-based modulations are commonly used, while some use sub-GHz bands as well in search of less interference and more signal integrity. Especially noteworthy are the IEEE 802.15.4-based protocols with spread spectrum (DSSS) [30] and LoRaWAN implementing it dynamically with its proprietary algorithm.

Other mechanisms include diverse medium access methods, such as the Zigbee beacons, synchronous protocols skipping events, such as BLE conn., the EnOcean “smart ACK”, which employs the acknowledgment to send data to the node, or the parent/friendship relationship of Zigbee and BLE, respectively, where the gateway or an extra node saves the messages directed to a node while this node stays in sleep phase (saving energy), for later retransmitting when it wakes up.

Regarding custom protocols addressing this niche market, to the best of our knowledge there has not being any attempt during the last decade to standardize or disseminate a WCP which solves the issues described above. Nevertheless, the literature focuses on improving the current protocols, proposing new features or solving vulnerabilities. For instance, Zhang et al. [31] and Laurentiu et al. [32] reported security flaws in BLE and LoRaWAN, Sigfox and NB-IoT, respectively. Mao et al. [33] proposed a security configuration strategy for IEEE 802.15.4 that adapts its level depending on the network security threats, service requirements and harvested energy. Meka and Fonseca [34] introduced a procedure that improves the Zigbee route selections through a different route-cost calculation. To conclude, among many other successful examples, Bomfin et al. [35] proposed an extension for the LoRa modulation which leads to a more energy-efficient system.

### 1.2. Contribution

In this work, we propose a new WCP intended for the harshest EH scenarios of IoT, with the latter goal of public standardization and broad expansion. *treNch* solves the ULP wireless sensor network (WSN) use cases through the use of a light cross-layer architecture with asynchronous transmissions, synchronous (subsequent) and optional receptions, short frame sizes and by a dynamic operation adaption depending on the energy status. This cross-layer principle is reflected in the proposed energy-management algorithm that uses two different operation modes for the sleep phase depending on the power input.

For evaluating our design, we conduct a detailed experimental analysis of *treNch* under different realistic scenarios, comparing the results with BLE at the same conditions and for its different profiles. Our focus is the power consumption at the nodes, but we discuss other features as well, such as the security, latency or reliability, among others. In particular, we propose techniques to implement low-overhead secure mechanisms that reduce the packet size without decreasing the security level, such as shorter over-the-air counters.

The main features that make *treNch* succeed in comparison with the state-of-the-art WCPs mentioned before are outlined as follows:

1. Power management algorithm sensing the energy status (cross-layer).
2. Control in the nodes, responsibility in the gateway, complexity in the client.
3. Asynchronous transmissions, synchronous (subsequent) and optional receptions.
4. Low frame overhead (including optimized security).
5. Randomized and controlled medium access (without sensing).
6. No physical-layer definition: adaptive to conditions.
7. Dynamic nodes emitting power.

The remainder of this article is organized as follows. In Section 2, we provide an in-depth description of the new WCP and its operation. In Section 3, we analyze the performance achieved through a comparison with BLE and finally, Section 4 draws the main conclusions of this work and expounds the focus for future work.

## 2. “*treNch*”

*treNch* is a cross-layer WCP and OS designed for ULP conditions in the IoT area, meant to be used with embedded EH systems as part of an interconnected WSN.

**Figure 2:**
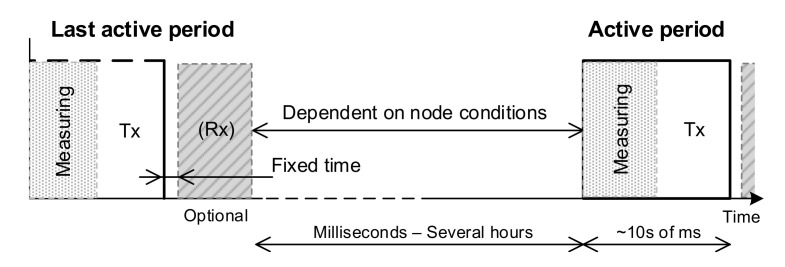
*treNch* principle of operation.

The operation principle is based on the node best-effort: Their transmissions are carried out asynchronously around a defined period, while the receptions (optional and previously notified) are performed synchronously right after the transmission (see Figure 2). In this way, a node transmits when its energy level allows it, not being forced to wake up to transmit/receive periodically by the risk of the connection being closed due to inactivity.

### 2.1. Network and Roles

**Figure 3:**
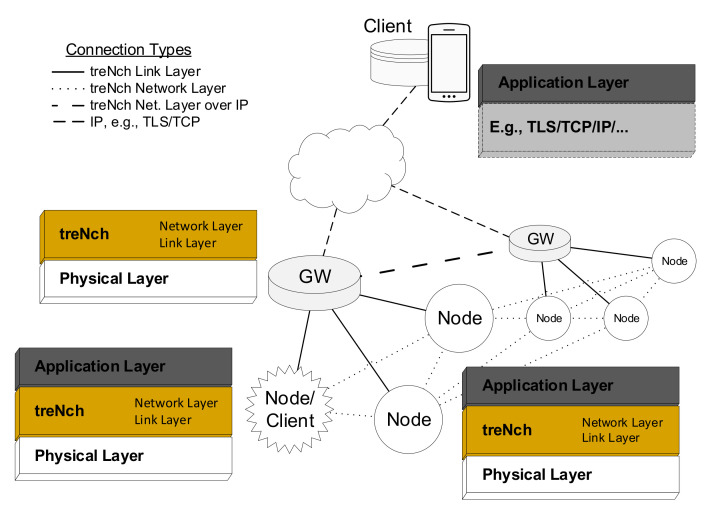
*treNch* network and protocol stack per device.

In *treNch*, a network is formed by subnetworks in star topology; see Figure 3, where new nodes have to perform an automatic registering process in order to be part of it. The communication is connectionless, closed and private.

A network has three different device roles:

- Node: Usually a sensor running under ULP conditions.
- Gateway (GW): Always ready to receive the node messages and answer them if pertinent. With network/link-layer functionalities, but transparent at application level. It defines a sub-network. No power limitations.
- Client (back-/frontend): Server and data consumer and in direct communication with the gateways. Interprets/triggers the application services, but with no network/link-layer functionalities. It approves new nodes registrations. No power limitations.

Subnetworks (gateways) and clients are interconnected through an auxiliary communication protocol not defined by *treNch*. Nonetheless, secure standards which are widely spread as TLS are recommended.

### 2.2. Protocol Stack

At link-layer level, a node is exclusively connected with a gateway through a bidirectional communication link. In the general use case, the gateway solely forwards the information coming from a node to the client and vice versa, apart from the network/link-layer directives, as the registering, acknowledgment or reception cycle, among others. This communication happens in the packet header but also in the payload with reserved parameters (see Section 2.3 and Section 2.4).

However, network-layer level direct node-to-node/s communication is also allowed through the gateway, by means of “channel subscription” (permanent receptions) or momentary point-to-point transmission. A *channel* is defined by a parameter class and a specific origin node. This is configured by the client in both cases, since its functionality is defined at application level.

To this end, the gateway keeps a queue of parameters for each node, sending them together in the payload of the next node reception phase. In the event of inter-sub-network transmissions, the gateway of the emitting node forwards the payload to the gateway owner of the destination node through a broadcast over the auxiliary protocol, e.g., IP.

Following this principle, a node without power concerns could also work as a client, as seen in Figure 3, with no need for the auxiliary protocol.

Regarding the physical layer, *treNch* does not define it in the protocol stack, leaving it open for every use case conditions. In this way, the noise, path losses, interferences of the environment, energy conditions and available hardware can be considered.

### 2.3. Frame Format

Table 2 and Table 3 introduce the frame formats for the two options within the bidirectional communication (node-to-gateway and gateway-to-node, respectively).

The frame encapsulates the packet structure with the *length* and *cyclic redundancy check (CRC)*. The physical-layer *preamble (preamb.)* needed by the radio is not defined, while the *sync word* is used as address. In the node-to-gateway transmissions, it indicates the node *origin ID*, whereas in the opposite case, it points out the node *destination ID (destin. ID)*. Since at link-layer level it is the only communication allowed; there is no need to include more address fields.

The packet is concluded with the *protocol version (prot. ver.)* and *CTR (treNch control)* fields. As seen in Table 2 and Table 3, the *CTR* follows two structures depending on the direction, including four different subfield types (functionalities explained in Section 2.4):

- *RX-Cycle*. It indicates after how many operation cycles the node will perform a reception. 0 forces the gateway to answer or, sent by the gateway, asks the node to receive again.
- *Reset*. It signals that the node is coming from a brown out reset (1), giving valuable information about its energy conditions.
- *ACK*. It acknowledges the last node reception.
- *RSSI*. It gives instructions to the node about the required emitting power, adapting it according the needs.

The payload is composed of an array of “*params*”, where a *param* is a group of two subfields: *type* and *data*. The *data* subfield carries the effective payload to transmit, while the *type* describes it with the data *class* and its *length*. Although some *classes* are reserved for the protocol, they do not follow any standardization, being defined by the user.

The bit assignation for every field sets the maximum *param-data* size in 7 Bytes and the maximum payload size in 27 Bytes. This gives a maximum theoretical throughput of 85.2%.

A message from a node is by default directed to the client and vice versa (only network configuration messages stay in the gateways). Nonetheless, the node-to-node communication use cases require special handling. In these scenarios, the header remains unchanged and the network recipient/origin is explicitly indicated with an address *param* in the payload, being the following *params* part of that connection. The payload, therefore, must be sequentially read. In this way, e.g., a node sending a packet with information to the client and to two different nodes would fill the payload with: client data *params*, address 1 *param*, node 1 data *param*, address 2 *param* and node 2 data *param*, in that order.

### 2.4. Workflow

**Figure 4:**
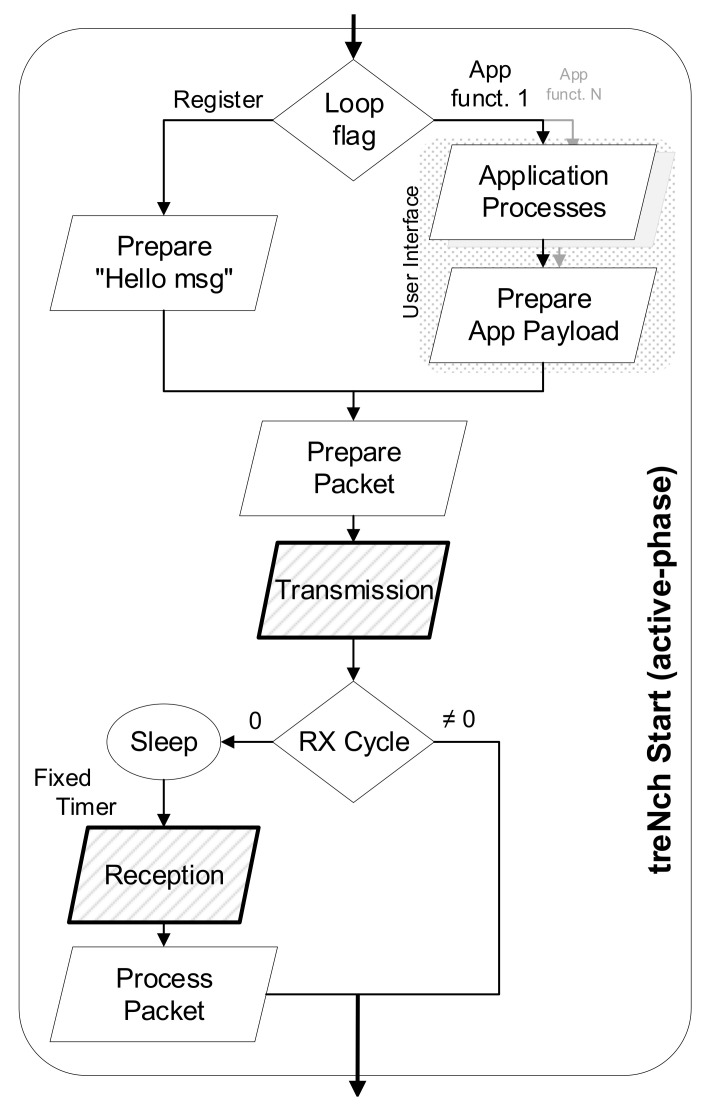
*treNch* node active phase workflow. Executed cyclically before and after a low power state (sleep).

Figure 4 represents the nodes active phase workflow. When a node enters *treNch_Start* (active phase), the different application profiles are executed, for later continuation to a transmission. In case the node is not registered yet, it proceeds first to registration (see Section 2.4.1).

Once the transmission is made, depending on the *RX-cycle*, the node executes the reception routine or goes directly to a low power consumption state for later waking up and starting the cycle again. The reception routine includes a sleep phase during a short and fixed time period, the packet reception and its processing at application- and network/link-layer levels.

The gateway workflow consists of a listening interface for the *treNch* node packets. It answers them with the data queue for each node when the *RX-cycle* indicates it and forwards the *params* to other nodes (queue) or through the auxiliary protocol interface to the clients or other gateways. Similarly, it includes forwarding the packets in the opposite direction or special actions regarding the network configuration, such as the registering or channel subscription, both described in the following.

#### 2.4.1. Registering

Before a node can interact with a network, it must become part of it through registering. A node sends a *“Hello message”* until a gateway answers with the registration information, starting only then with its application functionality.

The *Hello message* includes the node hardware ID, type and application. The reply includes the same hardware ID and the *treNch* ID, a shorter identifier (2 Bytes) that will be used as address for the node. Up to this time, when the *treNch* ID is assigned, the address employed in the frames (origin and destination ID) is a reserved one for broadcasting.

The process concludes when the client approves the registration, while the node remains in a quarantine list in the gateway with limited functionality (although transparent for the node).

In the case of a network with more than one gateway, every gateway broadcasts first to the rest of them the hardware ID, the received RSSI and a random number (for ties). The gateway with the best conditions adds the node to its sub-network and continues the process.

#### 2.4.2. Acknowledgments

*treNch* carries out acknowledgments at the link-layer level, not performing automatic re-sends (in the nodes) but notifying the application about incorrect transmissions. It is up to the application to decide if the message should be re-sent or ignored. In the case of the gateway, the retransmissions are automatic.

For transmissions in gateway-to-node direction, the correct reception is flagged with the *ACK* bit of the frame in the next cycle. For the opposite direction, the mere reception of a frame indicates implicitly the successful previous transmission, since it is the node who forces the gateway to send a packet. That means that a node application can request at any time an acknowledgment setting the *RX-cycle* to 0.

#### 2.4.3. Node-to-Node Transmission/Channel Subscription

First, the client triggers the process (application level), since it is the one with the overall information. It sends a command to a node, setting the transmission/reception of a *param-class* (see Table 2 and Table 3) to/from an specific node with several repetitions, a expiry date or certain circumstances as trigger. This might also change some other configuration parameters, such as the operation or reception periods according to the application. This process must be consistent with each node involved, since their applications have to be able to send/process a specific *param-class* as well as be realistic with the energy needs to fulfill the QoS.

The node then answers the command, acknowledging the client positively if applicable. Next, for the node-to-node transmission, it modifies at application level the destination of the messages for the following transmissions, indicating it on each frame. For the channel subscription, it sends with the same acknowledgment packet a network command to the gateway to set up the channel. Hereinafter, the gateway will forward automatically the configured *param-class* coming from the specified origin to the target node.

### 2.5. Power Management Algorithm and Energy Flag

The energy flag is an external binary signal informing about the status of the power capacitor/battery. It comes usually from the dc/dc converter required by the EH stage or even from the µC itself. The voltage levels between its thresholds for changing the value (with hysteresis) must encompass the worst case of the active phase energy needs and be above the minimum µC operation level.

**Figure 5:**
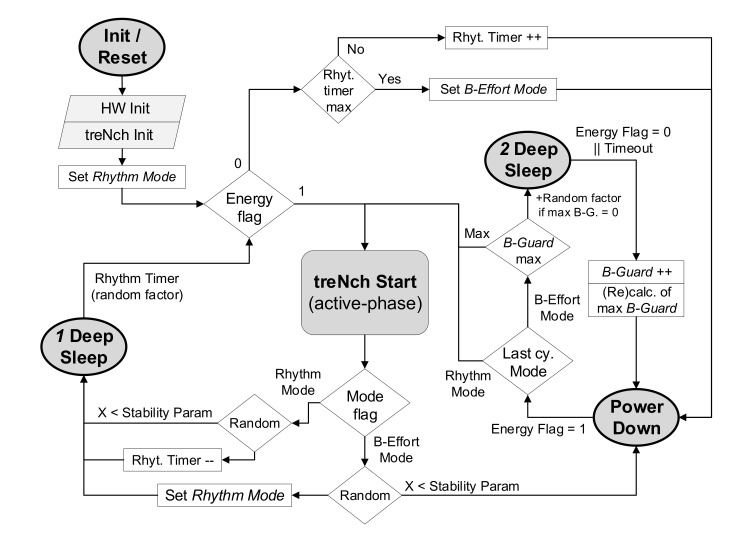
*treNch* node Sleep Modes and energy optimization algorithm workflow.

After initiation or a reset, the hardware and the *treNch* directives are initialized; see Figure 5. The last one includes setting up the protocol configuration and the registering information stored in flash, if this applies. By default, the system follows the *Rhythm Mode*. This means entering after the active phase in a deep sleep state to be woken up by a timer with the *minimum cycle time*. This value is defined by the user and has a slight random factor to avoid continuous collisions.

While the energy allows it, the system stays in this mode. In case of a decrease in the power input (energy flag down at the wake-up), *treNch* first increases the timer and goes to power-down state. Once the capacitor is charged (energy flag high), it wakes up and directly enters the active phase. In the next cycle, it goes again to deep sleep, with the updated timer value. If after several rounds the rhythm timer reaches its maximum (set by default at 115 of the minimum), the system goes to *B-Effort Mode*.

*B-Effort Mode* largely decreases the power consumption (see measurements of Section 3.1), since it sends the µC to power-down state after each active phase. With no timer but a wake-up by the energy flag (high), this allows the nodes to operate under extreme energy-adverse circumstances, in the range of up to 400 nW and with high power input fluctuations.

To avoid the violation of the *minimum cycle time* in the *B-Effort Mode*, *treNch* performs an estimation of the power input to set an approximation of a period without the wake-up timer. This is done with a second deep sleep state set with a clock that wakes up with the fall of the energy flag. The period is estimated with the time spent until the flag falls, knowing the sleep states power consumptions (µC data-sheet) and the energy needed for an active-cycle (value known by the user for the capacitor dimensioning and energy flag hysteresis configuration). The desired cycle time is achieved repeating the process according to the calculation until the maximum *B-Guard* value is reached, see Figure 5.

In the case of a maximum *B-Guard* resulting in less than one, the next operation cycle goes directly to the active phase (pure best-effort), performing randomly the estimation again. In the worst scenarios, this avoids a much bigger cycle time and waste of energy, due to the *B-Guard* calculation round.

*treNch* implements a procedure for returning cautiously to *Rhythm Mode* and to the *minimum cycle time*, through random attempts within the workflow. The periodicity of these attempts is configurable through the *stability parameter*, which incorporates in the method the expected power input fluctuation (set by the user).

The cross-layer energy management and usage defined by *treNch* achieves consequently great ULP operation while keeping the QoS, with brown out resets happening only in severe unexpected conditions.

### 2.6. Security

The existence of nodes with a very tight energy budget makes it complex to design a security concept without adding a significant overhead. Nevertheless, the criticality of security is such that compromises need to be introduced very carefully. We define four security levels:

- Level 0: no security. Packets are neither encrypted nor authenticated. For evaluation purposes. Not recommended for productive environments.
- Level 1: authentication. All packets are authenticated. For scenarios in which the exchanged data can be left public.
- Level 2: encryption and authentication. All packets are authenticated and encrypted.
- Level 3: encryption and authentication with extended *Message Integrity Check (MIC)*.

The secure modes have the following features:

#### 2.6.1. Secure Device Provisioning

Nodes are programmed with a pre-shared commissioning key, shared with the gateway and used only during the registering. Alternatively, it may also use an out-of-band mechanism. The goal is to securely distribute the node ID and a newly generated communication key to a node, for the first commissioning or after the node goes back to an unregistered state (e.g., after too many unsuccessful communication attempts with the gateway). For this, we propose using a commissioning key, which is reserved for the registering, together with a 104 bits random value, acting as a nonce for the cryptographic engine and included in the *Hello message* generated by the node. The gateway stores the nonce used by the node during the registering request and replies with the {communication key, node ID} pair. It uses the nonce chosen by the node with its most significant bit toggled (to avoid nonce repetition) and the pre-shared commissioning key to authenticate and encrypt the response. Commissioning exchange is always done with security level 3.

#### 2.6.2. Separation of Concerns

The gateway, during the registering, assigns different communication keys for the point-to-point communication with the different nodes, so if a communication key is exposed, only a particular link with a particular node is compromised.

#### 2.6.3. Replay Attack Protection

Replay attacks are avoided during registering, since the gateway stores all the random 104 bits nonces ever used by the nodes with a particular commissioning key and silently rejects further commissioning attempts using the same nonce. 104 bits nonces are sufficiently large, and registering attempts are sufficiently rare that the probability of the node randomly repeating a nonce is extremely low. Replay attacks are also avoided after registering, since the nodes always employ an increasing 104 bits counter, acting as a nonce, of which only the least-significant byte is added in the packet. The most significant bit of the counter is always used as zero by the node. If the gateway needs to reply, it will always use the last nonce received by the node, with the most significant bit set to one. If the gateway receives a counter equal to or lower than the last received counter, it will assume that a counter overflow occurred and will try to decode the packet adding one to the hidden (not transmitted over the air) most significant part of the counter. Gateways always store the last counter received from every node, while nodes only need to store in flash when the hidden most significant part is increased (e.g., every 255 packet transmissions). A loss of sync in the counter would be detected by the node, since no answer from the gateway would be received, and the node would react by going back to the unregistered state and trying a new commissioning attempt, which triggers a renewal of the key and reinitializes the associated counter. It might happen if an overflow is missed by the gateway (e.g., more than 255 successive packets are lost by the gateway) or if the node fails to properly store the most significant part of the counter in the flash (e.g., due to energy constraints).

In security levels 1 to 3, the whole packet is authenticated. The header is extended with the security level and counter fields and the *MIC* is appended before the CRC. In security levels 2 and 3, only the payload and the *CTR* field are encrypted (authenticated encryption with associated data).

In security level 1, *CBC-MAC* [36] authentication is proposed to generate the *MIC*, while in levels 2 and 3, *AES-CCM* [37] is proposed to get both authentication and encryption, using 128 bits keys and the AES-128 block cypher. *MIC* is truncated to the least-significant 4 Bytes in levels 1 and 2, and to the least-significant 8 Bytes in level 3. The length of the *MIC* field dictates how often a gateway or a node may trigger a key refresh. To avoid birthday attacks in levels 1 and 2, the key should be renewed before it has been used $2^{16}$ times, while in level 3 it should be renewed before it has been used $2^{32}$ times.

### 2.7. Other Features

#### 2.7.1. Reliability

*treNch* offers acknowledgments if the application demands it, thus, the reliability of the network ultimately resides in the energy conditions of the nodes and their predictability (best-effort).

#### 2.7.2. Latency

A node is configured with a *minimum cycle time* and, while the power input allows it remaining in *Rhythm Mode*, it operates asynchronously with that duty cycle up to a 15 higher value. If the power input decreases, the system enters in *B-Effort Mode*, where an estimation of that period is performed and ultimately follows the best-effort principle. The boundary case is where the *minimum cycle time* is set to 0, meaning this pure best-effort or operation by external interruptions.

#### 2.7.3. Medium Access Control

The short frame size, the high typical operation period for EH systems and the randomness of the transmissions act as the medium access control mechanism; together with the above mentioned minimum period of activity (avoiding that a node saturates the medium when the power conditions are favorable). The packet losses because of medium access collisions are consequently expected to be irrelevant [38].

#### 2.7.4. Emitting Power

In the *CTR-RSSI* field, the gateway gives instructions to the node in every reception to adjust the emission power. This helps to decrease the power consumption when the path losses are low or to reduce the wrong transmissions and increase the range in the contrary case.

#### 2.7.5. Hardware

Concerning the hardware requirements, in the case of a node, the mentioned energy flag needs an external binary signal informing about the energy level, i.e., a dc/dc converter or µC with that functionality. Moreover, the dimensioning of the capacitors must be realized together with the expected energy availability, device power consumption and the energy flag hysteresis [5].

In the case of a gateway, simply enough memory and computing power for fulfilling the above described activities are required.

## 3. Comparison with BLE

In this section, we analyze the performance of *treNch* through a detailed comparison with one of the main ULP protocols. We selected BLE considering not only its power consumption characteristics (one of the lowest, see Table 1), but also its wide spread and versatility within different scenarios. Hence, we analyzed BLE in connection, advertising and mesh low power modes.

The focus of the analysis is given to the power consumption of the nodes, since the main purpose of *treNch* is to address the requirements given by EH scenarios. Nonetheless, we also discuss other relevant aspects.

### 3.1. Power Consumption Analysis

The experiment was carried out with the first release of *treNch* in accordance with the characterization made in the previous section. The tested code includes the complete workflow of a node described in Figure 3, Figure 4 and Figure 5 with a simple application returning a random number, and a lite version of the gateway for one sub-network administration. The security grade chosen was Level 0 for better performance isolation.

In the case of BLE, we used the S132 Softdevice version 7.0.1 of Nordic Semiconductor, which includes the three operation modes of interest. The application over the BLE stack was the same, changing accordingly for the studied scenarios, and the security was disabled as well. For that purpose, we used an adaption of the UART emulator with transmission characteristic set as *Notify* and reception as *Write without response*.

In terms of hardware, we used for both protocols Nordic nRF52 development kits [39] with SDK version 16. It consists of a board with an ARM Cortex M4 system on chip and a 2.4 GHz transceiver, including the antenna. We chose this option because the theoretical power consumption according to the data-sheet is within the required ULP range and because Nordic provides with the official BLE Softdevice, simplifying our development steps and allowing easy reproduction of the results.

In both cases, we used two devices, working as gateway, client and node, server, in the terminology of *treNch* and BLE, respectively, and a third one acting as *friend* only for BLE mesh. For *treNch*, the client role was played by a computer with an USB-UART interface to the gateway. Moreover, for BLE we employed a commercial sniffer in parallel for extra validation of the communication (without interference in the measurements).

For a proper comparison, we configured the radio transceiver of *treNch* with the same physical-layer characteristics of BLE. Additionally, the emitting power was set to 0 dBm in all the cases, except of the scenario where we analyze its impact.

**Figure 6:**
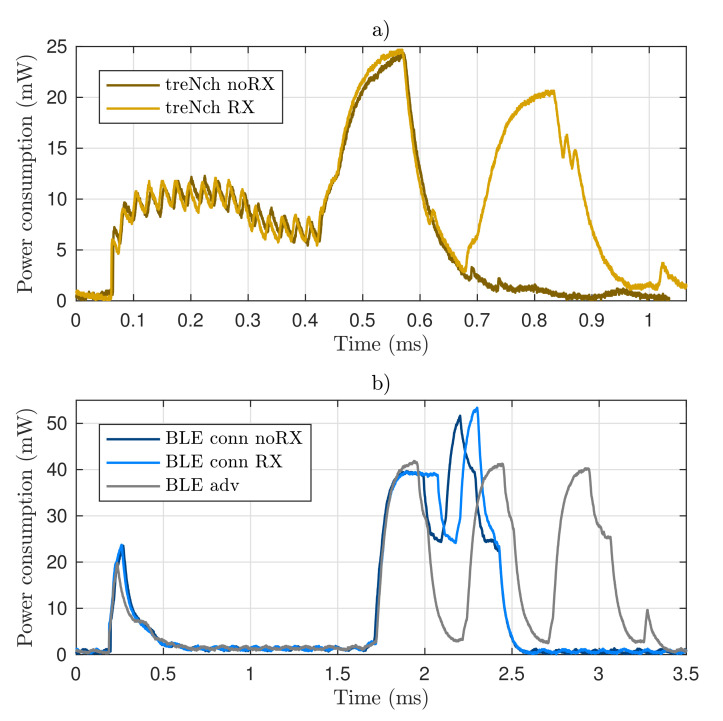
Measurement of power consumption in time during active phase. Most general cases of *treNch* (**a**) and BLE (**b**).

The measurements were carried out at 3 input power (given by the development kits) and with a Teledyne Technologies HD6054 oscilloscope for the active phases and an Agilent 34461A multimeter for the sleep phases. Figure 6 depicts an illustration of the measurements with a representation of the power consumption in time during the active phase for the most general cases of the protocols. The difference in frame sizes, processing time and peak power needed by the architectures can be clearly recognized. In particular, BLE already presents greater active-cycle times and a marked offset in consumption, seen during both the transmission and reception phases, due to the inherent software architecture processes.

In Table 4, the average power consumption and duration time of every event in the protocols are summarized. In each case, the effective payload was of 1 Byte (except for the large payload scenario), no security was used and an error- and noise-free channel was assumed.

After that, we evaluated different use cases where we compared the performance between *treNch* and the diverse BLE options, based on the characterized events. We join the corresponding event consumptions, satisfying the protocols workflow to determine their energy demands for every situation.

#### 3.1.1. Transmitter

In this scenario, the nodes do not perform application data receptions. We analyzed the average power consumption over a cycle in a broad period spectrum, assuming that the power input was always sufficient and uninterrupted for the required operations.

**Figure 7:**
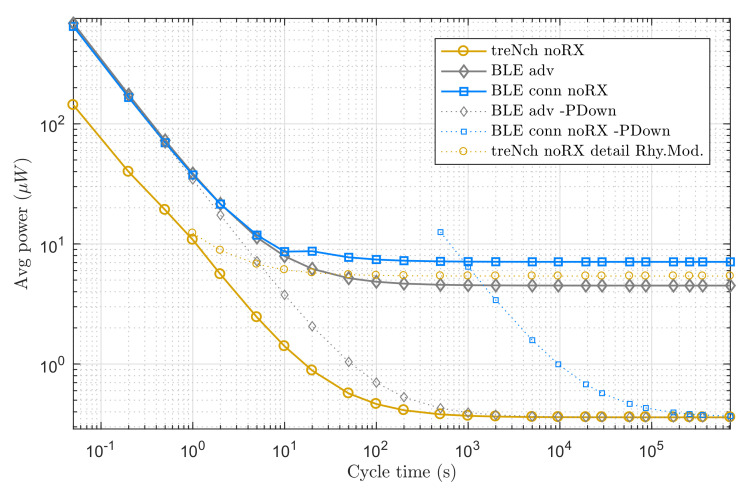
Use case of node with only transmission needs. Average power of a complete cycle over cycle time. *Note logarithmic scale in both axes*.

Figure 7 reveals how *treNch* stays always below in the cycle average power consumption, thanks mostly to the energy-management algorithm described in Section 2.5. The figure includes for more detail the *treNch* consumption in the most power-demanding situation of the *Rhythm Mode* (dotted line), meaning this pure timer operation during the sleep phase. For a given cycle time, when the power input is distanced from that curve, *treNch* starts operating in *B-Effort Mode*, with the period estimated from the power input. As soon as the power rises, *Rhythm Mode* takes over again with a more precise cycle.

BLE adv. (same case as mesh) attends to only one operation principle, being the power consumption dominated by the transmission energy in the first part of the graph (done in three different channels) and by the sleep phase in the second, with the transmission negligible and tending to constant.

For BLE conn., the curve has two tendencies. This is because of the obligation of sending keep alive messages as the operation cycle increases, to keep the connection open. The consumption then stays constant, tending to the sleep phase consumption too. In contrast to *treNch*, both BLE cases have a fixed and stable cycle time.

In the BLE cases, although not implemented by the BLE functionality, we tested how the consumption would be if the application would set the µC into power-down state, for being later woken up by an external signal (dotted lines in the graph). For that, we used the power-down value consumption of *treNch* as reference. As result, despite the sleep consumption decreasing greatly, only in cycle times higher than an hour is beneficial for BLE conn., since the inactivity during the power-down means losing the connection and opening it in every cycle, aside of the security issues that arise. Likewise, it is still not enough for BLE adv. to overtake *treNch*, due to the three-channel transmission. Notice that for this approach to follow a specific cycle time, an external clock not considered in the consumption would be needed, or the implementation of an algorithm similar to *treNch*.

#### 3.1.2. With Reception

**Figure 8:**
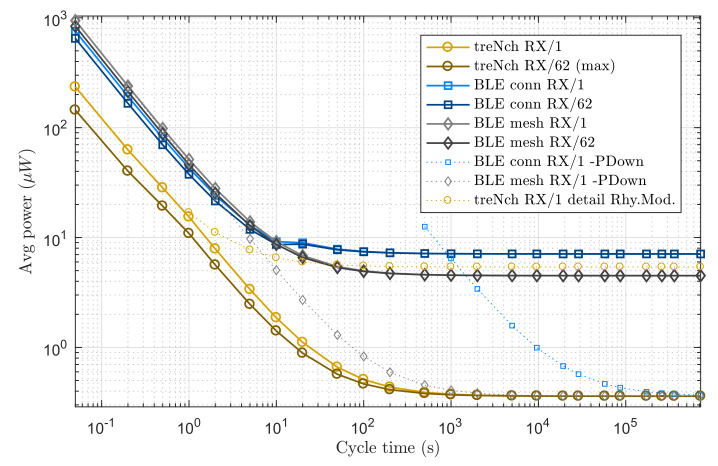
Use case of node with different reception period needs (every N active cycles: *RX-Cycle*). Average power of a complete cycle over cycle time. *Note logarithmic scale in both axes*.

This scenario presents nodes with application data receptions over the same period spectrum as the previous one and again assuming sufficient and uninterrupted power input. Figure 8 depicts the tested protocols with a reception in every active-cycle and for the maximum allowed by *treNch* (62, i.e., maximum given by the *RX-Cycle* frame field). We also evaluated the hypothetical use of the power-down state by BLE and, again for reference, the *Rhythm Mode* of *treNch*.

The results are very similar to the previous scenario. The power consumptions of BLE conn. and *treNch* do not vary significantly, since for both protocols the methodology is equal and the extra energy needed for the reception is comparable to some more time in sleep state. BLE mesh, however, presents a big difference respect to BLE adv. and even higher consumption than BLE conn. in the low cycle times. This is due to the polling and reception to the *friend* device in three different channels. Nevertheless, it surpasses quickly BLE conn., although not *treNch*, due to the 96 h friend poll timeout and the lower sleep state consumption, where all finally tend.

#### 3.1.3. Critical Energy

This use case assumes the worst situation where the power input allows only the active phase. We consider that the µC is turned off shortly after the end of every active cycle due to lack of energy and the sleep consumption, with the sleep phase having no effect in the analysis. This evaluation also contemplates the occasional situation after a brown out, often happening with EH devices.

Table 5 presents the results with and without reception. The consumption of BLE conn., 2 orders of magnitude above *treNch*, proves how highly inefficient the synchronous protocols are in this kind of scenarios, because of the need for re-connecting. BLE adv./mesh stays from 1 to 2 orders of magnitude above as well, due mostly to the initialization and guard time and in the reception case to the three channels polling at the *friend* device.

#### 3.1.4. Distance

In this scenario, we evaluated the energy needed for an active-cycle depending on the distance to the receiver. Again, the power consumption during the sleep time is not contemplated and we considered that the nodes are coming from a normal wake-up, not a reset (it would add a fixed energy consumption because of the startup). We analyzed for each protocol the case with and without reception.

For the analysis, we calculated the distance as the theoretical maximum range achieved according to [40]:

(1) pathloss=40+25×logd,

with *d* the distance, assuming an isotropic antenna, a channel without obstacles, noise or reflections and given the sensitivity of the used hardware of −93 dBm.

**Figure 9:**
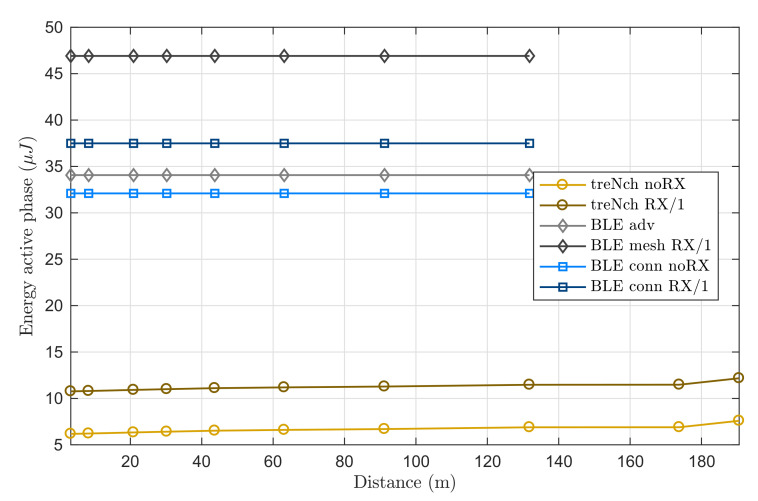
Use case of node at different distances from the gateway. Energy of active phase (no sleep time considered) over distance. Notice that BLE remains constant.

Aside of the energy differences in the transmission cycle for the general case (due to the mandatory master/slave reception in BLE conn. and to the three-channel transmission in BLE adv. and mesh), BLE does not implement automatic emitting power adjustment apart from the power class election. Thus, as Figure 9 illustrates, their curves remain constant (0 dBm transmission). *treNch*, on the other side, provides feedback to the nodes depending on the received signal strength by the gateway (*CTR-RSSI* field), achieving in this experiment up to a 8 times lower energy consumption and a greater range (bounded in the graph by the minimum and maximum emitting power of the used hardware, −40–4 dBm).

#### 3.1.5. Large Payload

This use case analyzes the consumption of an application with large payload requirements. Since *treNch* does not implement data fragmentation in the current revision, we considered the maximum payload size admitted for each protocol within one single frame, i.e., *treNch*, 27 Bytes; BLE conn., 23 Bytes and BLE adv./mesh, 26 Bytes.

**Figure 10:**
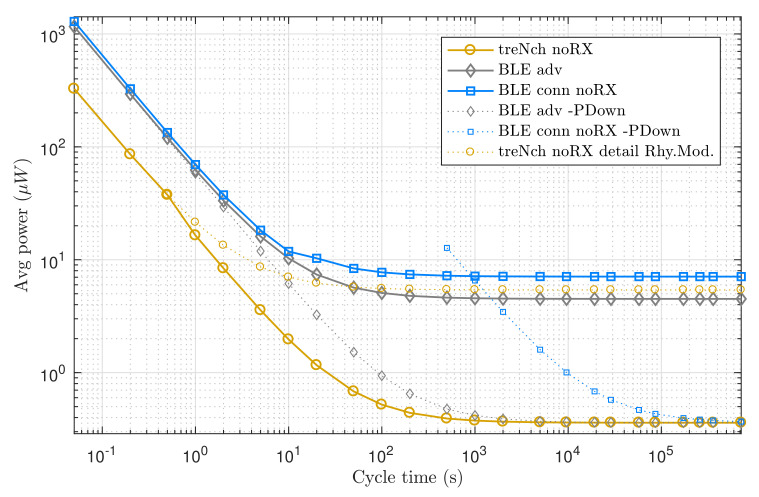
Use case of node with large payload needs. Average power of a complete cycle over cycle time. Maximum payload size for every WCP: *treNch*, 27 Bytes; BLE conn., 23 Bytes and BLE adv., 26 Bytes. *Note logarithmic scale in both axes*.

The results, Figure 10, are very similar to the ones obtained in Section 3.1.1 (1 Byte of effective payload). The consumption gets increased around twice its value in every protocol at low cycle times, although remains dominated by the sleep phase, thus tending for higher cycle times to the same figures.

The larger payload size makes the existing differences in the frame overhead close to irrelevant. However, this matter is not enough to overcome the lighter architecture and processes of *treNch*, as reflected in Figure 6. Table 4 details the great contrast in the power and time demands of these events.

### 3.2. Other Aspects

#### 3.2.1. Error Handling

After an error with a packet transmission, BLE conn. always re-sends it due to the layer-link intrinsic acknowledgments, normally in the next *connection interval*. This might be valuable in some applications, but it can also cause unnecessary overhead, delay and energy waste, in scenarios where an old message is outdated and no longer desired. BLE adv. operates in the opposite boundary: without possibility of acknowledgments. *treNch*, contrary to this, gives the opportunity of deciding to the application, also in the next cycle. This principle is shared with BLE mesh.

#### 3.2.2. Latency

The average latency in BLE conn. in both directions is half the *connection interval*, being a complete cycle the worst case (synchronous). In the rest of BLE modes and *treNch*, an interruption in the node can trigger the transmission in any moment (asynchronous). In the opposite direction (not including BLE adv.), the latency cannot be predicted since it depends on the nodes.

#### 3.2.3. Reliability

Giving the analyzed protocols the acknowledgment option (except BLE adv.) and using in the experiment the same physical layer, the differences in reliability come by the benefit of the BLE conn. channel hoping algorithm and the triple transmission of BLE adv. and mesh, ensuring more robustness. *treNch* deals with noisy environments leaving the physical-layer characteristics open for every use case. Moreover, its best-effort principle and light architecture allow it to keep functioning under extreme lower power conditions.

#### 3.2.4. Topology

Only BLE mesh supports the bidirectional communication between nodes as *treNch* does. Neither BLE conn. considers this topology only achievable at application level; nor BLE adv., which broadcasts unidirectionally.

#### 3.2.5. Interoperability

One of the main features of BLE is its interoperability through standardized profiles (*services*) and *characteristics*. *treNch* seeks for more simplicity leaving to the application all the responsibility and setting up independent networks, which may operate even at different frequency bands.

#### 3.2.6. Security

The total overhead of the packet by adding security is: 2-bits, to specify the security level; 1 Byte to specify the counter, and either 4 Bytes (security level 1 and 2) or 8 Bytes (security level 3) for the *MIC*. In comparison, the counter used in BLE is 3 Bytes long, since it transmits a larger part of the nonce over the air and in BLE Mesh the packet is authenticated and encrypted at two different levels (network and application), so two different *MIC*s (either 4 or 8 Bytes long each) are required.

## 4. Conclusions

This paper has introduced *treNch* for use in EH ultra-low-power WSN. We described its operation principles and compared it with the BLE modes in realistic ULP scenarios, demonstrating a better performance in each of them. Its proposed light architecture with asynchronous transmissions, synchronous and optional receptions, short frame sizes and the unique energy-management algorithm, make *treNch* achieve power consumptions from 1 to 2 orders of magnitude lower than BLE.

The proposed energy-management algorithm, exploiting the cross-layer paradigm in the WCP, entails a large advance concerning the power consumption without losing QoS. It achieves best-effort communications with defined periodicity control, without wasting energy in timers during the sleep phase. In addition, for more demanding applications, the switch to more reliable communication is carried out automatically, as soon as the energy conditions are favorable.

As a result of the described features, we set a new minimum operation threshold for the power input, opening the possibility for new EH scenarios within the IoT, where the application can operate automatically from pure best-effort to nearly real time, depending on the environment.

The experimental results have proven as well that the synchronous WCP as BLE conn. are highly inefficient for EH use cases, where the energy flow is not assured, neither predictable in most of the scenarios. On the other side, giving the control to the nodes for acting according to their needs, stands as a more efficient practice, since it does not waste energy in tedious protocol procedures. BLE adv. and BLE mesh advance in this direction too. Although BLE adv. does not implement receptions, nor security option, and BLE mesh, more focused on the mesh nodes than in the low power ones, resolves it in a more burdened way.

We also proposed a security scheme that uses standard and proven mechanisms and are straightforward to integrate, in addition to being seamless enough that it does not increase the processing time (assuming most transceivers have an AES accelerator) or the frame size significantly, i.e., the power consumption.

In other respects and future work, we envision an out-of-band registering process, over NFC/RFID or a low-frequency receiver, enabling the use of radio transmitters (hardware without reception capabilities). This would boost the use of low-cost nodes, without compromising privacy and security. Moreover, we plan to design data fragmentation, currently only possible at application layer.

## Figures and Tables

**Table 1:** Most common ultra-/low power WCPs.

	Power Consumption	Range	Topology	Data Rate	Standard	Power Management Techniques
*BLE* [14,18,19]	Ultra-low 10 s of µW	WPAN 100 m	Star, Bus, Mesh	1 and 2 Mbps	Bluetooth SIG	Phy.-layer (FSK-based mod.) Periph. skip connections Mesh: Friendship, flooding
*Wi-Fi* [14,18]	Low power 10 s of mW	WLAN 250 m	Star, Mesh	11–300 Mbps	IEEE 802.11	-
*Zigbee* [14,18,19]	Ultra-low ∼50 µW	WPAN 100 m	Star, Mesh, Cluster	20, 40, 250 kbps	IEEE 802.15.4	Phy.-layer (O-QPSK mod., DSSS) Parent relationships Beacon-enabled/CSMA/CA
*Z-Wave* [14,20]	Low power ∼ µW	WPAN 30 m	Mesh, Star	40 kbps	Proprietary	Low data rate Phy.-layer (FSK mod., sub-GHz) Asynch. TX, Synch. RX
*ANT* [21,22]	Ultra-low ∼80 µW	WPAN 30 m	Star, Bus, Mesh	60 kbps	Proprietary	Phy.-layer (FSK-based mod.) Isochro. + Medium-sensing
*Thread* [20,23]	Ultra-low ∼50 µW	WPAN 100 m	Mesh, Star	250 kbps	IEEE 802.15.4	Phy.-layer (O-QPSK mod., DSSS) Asynchronous CSMA/CA
*EnOcean* [24,25]	Ultra-low µW	WPAN 30 m	Star	125 kbps	Proprietary	Phy.-layer (ASK/FSK mod., sub-GHz) Low overhead Asynch. TX, Smart ACK
*LoRaWAN* [26,27,28]	Low power 100 s of µW	WWAN 10 kM	Star	0.3–50 kbps	Proprietary	Phy.-layer (LoRa mod., sub-GHz) Low data rate Asynch. TX, Subsequent RX

WPAN, wireless personal area network; WLAN, wireless local area network; WWAN, wireless wide area network.

**Table 2:** treNch frame format for node-to-gateway transmissions (security Level 0).

PREAMB.	**ORIGIN** ID (SYNC)	LENGTH	PROT. VER.	PAYLOAD					**CTR**/_NODE_			CRC
				PARAM			PARAM 2	…				
				Type		Data			RX-Cycle	**Reset**	**ACK**	
				Class	Lenght							
X Bytes	2 Bytes	5 bits	3 bits	5 bits	3 bits	N Bytes	M Bytes	…	6 bits	1 bit	1 bit	2 Bytes
		1 Byte		1 Byte					1 Byte			

**Table 3:** treNch frame format for gateway-to-node transmissions (security Level 0).

PREAMB.	**DESTIN.** ID (SYNC)	LENGTH	PROT. VER.	PAYLOAD					**CTR**/_NODE_		CRC
				PARAM			PARAM 2	…			
				Type		Data			RX-Cycle	**RSSI**	
				Class	Lenght						
X Bytes	2 Bytes	5 bits	3 bits	5 bits	3 bits	N Bytes	M Bytes	…	6 bits	2 bits	2 Bytes
		1 Byte		1 Byte					1 Byte		

**Table 4:** Measurements of power consumption for every event.

Event	Duration (ms)	Avg. Power (mW)
*treNch*		
Start, TX (0 dBm)	15.7	3.9
TX (from DSleep)	700 × 10^−3^	9.8
TX (from PDown)	819 × 10^−3^	12.7
TX Max. Pay. (27 B)	1.5	10.7
+ RX	1.1	4.2
Registering Cycle	15.7	4.9
Deep Sleep	-	5.4 × 10^−3^
Power Down	-	360 × 10^−6^
***BLE***		
Conn.-TX (0 dBm)	2.6	12.3
Conn.-TX Max. Pay. (23 B)	2.7	18.9
Conn.-TX, RX	2.7	14.2
Conn.-Advertising, Pairing	5.6 × 10^−3^	1.1
Conn.-Keep Alive	2.4	11.0
Conn.-Sleep	-	5.4 × 10^−3^
Adv. - Start, TX (0 dBm)	441.8	778 × 10^−3^
Adv. & Mesh-TX	3.1	10.9
Adv. & Mesh-TX Max. Pay. (26 B)	3.8	15.2
Adv. & Mesh-Sleep	-	4.5 × 10^−3^
Mesh-TX, RX	4.2	11.1
Mesh-Start, TX	518.3	2.9

**Table 5:** Use case of node with critical power input.

	Energy of Active Phase (mJ)	
	No RX	With RX
*treNch*	61 × 10^−3^	66 × 10^−3^
*BLE conn.*	6.09	6.10
*BLE adv./mesh*	344 × 10^−3^	1.54
